# A nucleoside anticancer drug, 1-(3-C-ethynyl-β-D-ribo-pentofuranosyl)cytosine (TAS106), sensitizes cells to radiation by suppressing BRCA2 expression

**DOI:** 10.1186/1476-4598-10-92

**Published:** 2011-07-28

**Authors:** Shunsuke Meike, Tohru Yamamori, Hironobu Yasui, Masato Eitaki, Akira Matsuda, Masami Morimatsu, Masakazu Fukushima, Yasundo Yamasaki, Osamu Inanami

**Affiliations:** 1 Laboratory of Radiation Biology, Department of Environmental Veterinary Sciences, Graduate School of Veterinary Medicine, Hokkaido University, Sapporo, 060-0818, Japan; 2 Laboratory of Medicinal Chemistry, Faculty of Pharmaceutical Sciences, Hokkaido University, Sapporo, 060-0812, Japan; 3 Division of Disease Model Innovation, Institute for Genetic Medicine, Hokkaido University, Sapporo, 060-0815, Japan; 4 Taiho Pharmaceutical Co., Ltd., Chiyoda-ku, Tokyo, 101-8444, Japan

**Keywords:** radiation, DNA repair, homologous recombination

## Abstract

**Background:**

A novel anticancer drug 1-(3-C-ethynyl-β-D-ribo-pentofuranosyl)cytosine (ECyd, TAS106) has been shown to radiosensitize tumor cells and to improve the therapeutic efficiency of X-irradiation. However, the effect of TAS106 on cellular DNA repair capacity has not been elucidated. Our aim in this study was to examine whether TAS106 modified the repair capacity of DNA double-strand breaks (DSBs) in tumor cells.

**Methods:**

Various cultured cell lines treated with TAS106 were irradiated and then survival fraction was examined by the clonogenic survival assays. Repair of sublethal damage (SLD), which indicates DSBs repair capacity, was measured as an increase of surviving cells after split dose irradiation with an interval of incubation. To assess the effect of TAS106 on the DSBs repair activity, the time courses of γ-H2AX and 53BP1 foci formation were examined by using immunocytochemistry. The expression of DNA-repair-related proteins was also examined by Western blot analysis and semi-quantitative RT-PCR analysis.

**Results:**

In clonogenic survival assays, pretreatment of TAS106 showed radiosensitizing effects in various cell lines. TAS106 inhibited SLD repair and delayed the disappearance of γ-H2AX and 53BP1 foci, suggesting that DSB repair occurred in A549 cells. Western blot analysis demonstrated that TAS106 down-regulated the expression of BRCA2 and Rad51, which are known as keys among DNA repair proteins in the homologous recombination (HR) pathway. Although a significant radiosensitizing effect of TAS106 was observed in the parental V79 cells, pretreatment with TAS106 did not induce any radiosensitizing effects in BRCA2-deficient V-C8 cells.

**Conclusions:**

Our results indicate that TAS106 induces the down-regulation of BRCA2 and the subsequent abrogation of the HR pathway, leading to a radiosensitizing effect. Therefore, this study suggests that inhibition of the HR pathway may be useful to improve the therapeutic efficiency of radiotherapy for solid tumors.

## Background

Radiation is one of the effective treatments for cancer therapy. Double-strand breaks (DSBs) in tumor cells exposed to ionizing radiation are believed to cause apoptosis, mitotic catastrophe and reproductive cell death [1,2]. However, because DSBs are immediately repaired by DNA repair mechanisms, the cellular DNA repair capacity seems to be closely associated with the outcome of radiotherapy [3]. Therefore, targeting DNA DSB repair pathways can be a potential therapeutic strategy to enhance the antitumor effect of radiation.

In repair mechanisms for DNA DSBs, there are two major pathways, non-homologous end joining (NHEJ) and homologous recombination (HR). In the NHEJ pathway, which is active during all phases of the cell cycle, DNA ends are joined with little or no base deletion at the end-joining site. In contrast, the HR pathway employs the sister chromatid after DNA replication, which results in error-free repair. Therefore, HR is most active in the late S and G2 phases [4]. In the HR pathway, a large number of proteins are involved, including Mre11-Rad50-NBS1 (MRN) complex, RPA, Rad51, BRCA1, and BRCA2. In response to DSBs, Rad51 forms nucleoprotein filaments on single-strand DNA (ssDNA) and causes strand exchanges between ssDNA and homologous double-strand DNA [5]. Therefore, Rad51 acts as a central player in HR and its cellular expression level affects radiosensitivity and chemosensitivity [6]. BRCA2 phospholylated at Ser3291 directly interacts with Rad51 through BRC repeats, facilitating the formation of Rad51 filaments [7,8]. Accordingly, BRCA2 is a key protein to promote Rad51 recombinase function after DNA damage. In fact, cells lacking functional BRCA2 exhibit genomic instability and sensitivity to DNA-damaging agents such as etopside, bleomycin and X-rays [9,10].

**Figure 1:**
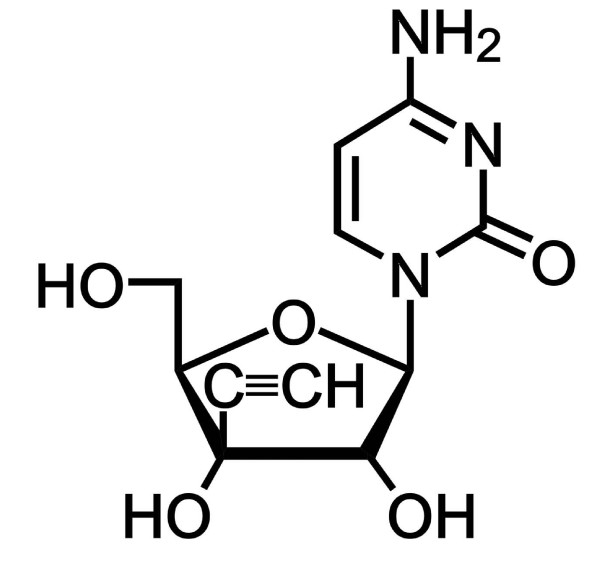
**TAS106**. The chemical structure of TAS106.

The ribonucleoside anticancer drug, 1-(3-C-ethynyl-β-D-ribo-pentofuranosyl)cytosine (ECyd, TAS106) inhibits RNA synthesis through competitive inhibition of RNA polymerase (Figure 1) [11]. TAS106 rapidly undergoes phosphorylation to a 5'-triphosphate form (ECTP) after its uptake into cells, and ECTP strongly inhibits RNA polymerase to cause RNA synthesis inhibition [12,13]. Furthermore, Naito *et al*. have demonstrated that TAS106 strongly induces JNK-dependent apoptosis through activation of an RNase L-mediated RNA degradation pathway [14]. In the phosphorylation of TAS106, uridine/cytidine kinase (UCK) is responsible for the first phosphorylation of TAS106 to the 5'-monophosphate form. The UCK activity in tumor cells is higher than that in non-tumor cells, thereby causing the accumulation of TAS106 preferentially in tumor cells [13-16]. We have previously reported that a sublethal dose of TAS106 strongly suppresses the expression of anti-apoptotic proteins and G2/M checkpoint-related proteins, and enhances radiation-induced cell death and growth delay in gastric tumor cell lines MKN45, MKN28 and murine rectum adenocarcinoma cell line Colon26 *in vitro *[17] and *in vivo *[18]. Furthermore, this radiosensitizing effect is also observed in radioresistant hypoxic cells through the inhibition of hypoxia inducible factor 1α (HIF-1α) expression [19]. However, the precise mechanism underlying TAS106-induced radiosensitization remains elusive.

In this study, to further examine the mechanism of TAS106-induced radiosensitization, we investigated whether TAS106 could modify the repair capacity of DNA DSBs. We demonstrate that TAS106 decreases cellular DNA DSB repair capacity and radiosensitizes human lung carcinoma A549 cells. In addition, we show that this radiosensitizing effect is mainly due to abrogation of the HR pathway through the suppression of BRCA2 expression.

## Results

### TAS106 enhances radiosensitivity in tumor and immortalized cells

**Figure 2:**
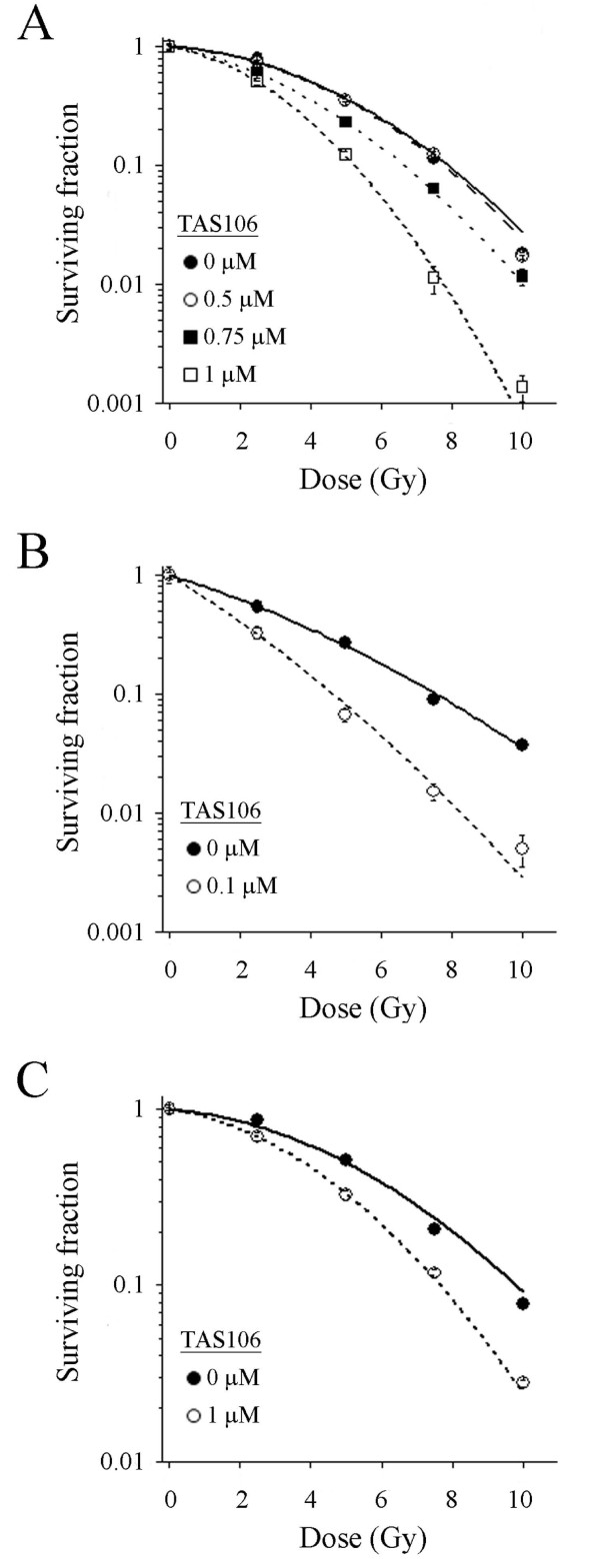
**Effect of TAS106 on radiosensitivity of A549, HEp-2 and V79 cell lines**. (A-C) Dose-response curves for cells exposed to X-irradiation with or without indicated concentrations of TAS106. After the pretreatment with TAS106, cells were irradiated and assessed for its radiosensitizing effect by measuring clonogenic cell survival. (A) Clonogenic cell survival for A549 cells. *Closed circle*; X-irradiation only, *open circle*; X-irradiation + 0.5 μM TAS106, *closed square*; X-irradiation + 0.75 μM TAS106, *open square*; X-irradiation + 1 μM TAS106. (B) Clonogenic cell survival for HEp-2 cells. *Closed circle*; X-irradiation only, *open square*; X-irradiation + 0.1 μM TAS106. (C) Clonogenic cell survival for V79 cells. *Closed circle*; X-irradiation only, *open square*; X-irradiation + 1 μM TAS106. Data are expressed as mean ± SE of three experiments.

To determine whether pretreatment with TAS106 enhanced the radiosensitivity of tumor and immortalized cells, we performed a clonogenic survival assay. Figure 2A shows the X-ray dose-response curves for cell survival in A549 cells pretreated with TAS106 at various concentrations. Pretreatment with TAS106 suppressed the clonogenic cell survival in a concentration-dependent manner and the 10% lethal dose (D_10_) of the surviving fraction was reduced from 7.88 Gy in the control to 5.24 Gy by the treatment with 1 μM TAS106. The sensitizer enhancement ratio (SER) judged by the D_10 _was 1.50, indicating the increase of sensitivity to X-irradiation induced by TAS106. In addition, HEp-2 cells and V79 cells pretreated with TAS106 also exhibited sensitization to X-irradiation as shown in Figures 2B and 2C. SER values for HEp-2 and V79 cells were 1.59 and 1.28, respectively. Furthermore, TAS106 increased the α values in all cell lines tested (Table 1). These results indicated that pretreatment with TAS106 enhanced radiosensitivity in various cell lines.

**Table 1:** Summary of survival curves parameters

Cell line	TAS106	α	SER_α_	β	SER_β_	D_10_	SER_D10_
	(μM)	(Gy^-1^)		(Gy^-2^)		(Gy)	
A549	0	0.04	-	0.032	-	7.88	-
	0.5	0.04	1.00	0.033	1.03	7.77	1.01
	0.75	0.12	3.00	0.033	1.03	6.73	1.17
	1	0.12	3.00	0.061	1.91	5.24	1.50
Hep-2	0	0.21	-	0.013	-	7.49	-
	0.1	0.41	1.95	0.017	1.31	4.70	1.59
V79	0	0.04	-	0.020	-	9.78	-
	1	0.07	1.75	0.030	1.50	7.67	1.28
V-C8	0	0.33	-	0.024	-	5.09	-
	1	0.42	1.27	0.020	0.83	4.51	1.13

The data were fitted using the linear-quadratic model, SF = exp(-αD-βD^2^). The 10% lethal dose (D_10_) was calculated from the α and β values. The sensitizer enhancement ratio (SER) was calculated from the each values with or without TAS106.

### TAS106 suppresses cellular DSB repair capacity

**Figure 3:**
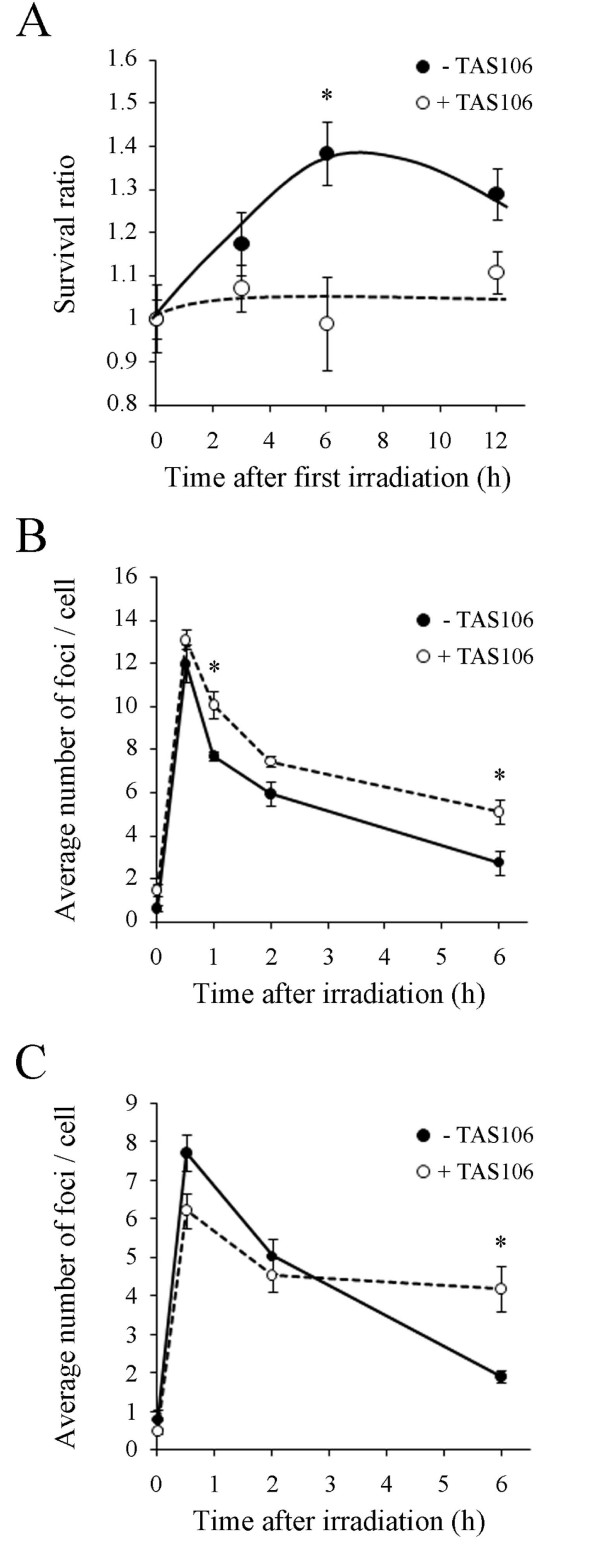
**TAS106 suppressed DNA repair capacity in A549 cells**. (A) Survival ratio of A549 cells after fractionated irradiation. Cells were pretreated with TAS106 (1 μM) for 24 h and then irradiated (2.5 Gy). After incubation for the indicated times, they were irradiated (2.5 Gy) again and cultured for colony formation. The survival ratios were normalized to unity at time 0 h for each group. Data are expressed as mean ± SE of three experiments. *p < 0.05, significant difference by the Mann-Whitney U test. (B) γ-H2AX and (C) 53BP1 focus formation after X-irradiation. Cells were pretreated with TAS106 (1 μM) for 24 h, followed by X-irradiation (1 Gy). They were then fixed at the indicated times to evaluate the nuclear γ-H2AX focus formation. The numbers of foci in at least 20 cells were scored and the average numbers were plotted in the graph. Data are expressed as mean ± SE of three experiments. *p < 0.05, significant difference by Student's *t*-test.

To investigate whether TAS106 radiosensitized tumor cells by inhibiting DNA DSB repair, we measured the sublethal damage (SLD) repair in A549 cells. The SLD assay is based on evidence that cell survival increases with extended interval times between two split doses of X-rays, provided that the cells can repair the initial DSBs prior to the second irradiation [20]. Therefore, SLD repair is considered to reflect the cellular DSB repair capacity. As shown in Figure 3A, the survival ratio in A549 cells exposed to fractionated irradiation increased as the interval time extended up to 6 h and then decreased at 12 h. The maximum increase of the survival ratio was 1.4 at the 6 h interval time. In contrast, there was little increase in the survival ratio of A549 cells pretreated with TAS106. This result suggested that the pretreatment with TAS106 suppressed DNA DSB repair in A549 cells.

To further support these data, we analyzed DSBs in X-irradiated cells by γ-H2AX and 53BP1 foci formation assay. Histone H2AX is known to be phosphorylated at serine 139 (γ-H2AX) immediately after DSB induction, and then γ-H2AX forms nuclear foci in the region of the DSBs, and subsequently undergoes dephosphorylation after the repair of DNA strand breaks. p53 binding protein 1 (53BP1) also localizes at the DSBs region. Therefore, the numbers of γ-H2AX and 53BP1 foci are used as a measure of the relative amount of DSBs and repair kinetics [21,22]. When cells were irradiated at 1 Gy, the average number of γ-H2AX foci per cell peaked at 30 min after irradiation and decreased with time for both cells with or without TAS106 (Figure 3B). There were no significant differences in the numbers of γ-H2AX foci between cells without and with TAS106 30 min after irradiation. However, at 1 h, 2 h and 6 h after irradiation, the numbers of foci in cells pretreated with TAS106 were higher than in cells without TAS106. In addition, the average number of 53BP1 foci in cells treated with TAS106 was significantly greater than that in cells without TAS106 at 6 h after irradiation. These results suggested that TAS106 inhibited DSB repair.

### TAS106 suppresses the expression of DSB repair proteins

**Figure 4:**
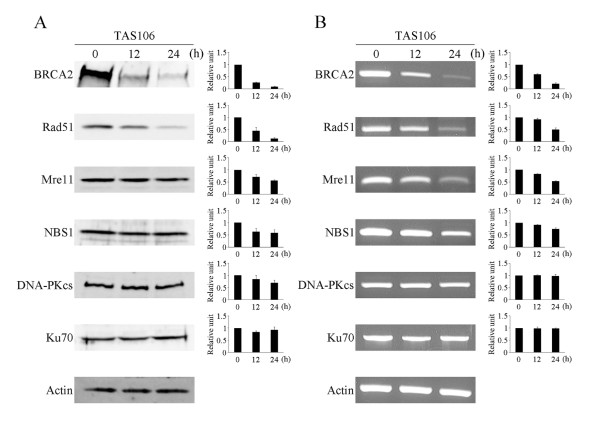
**TAS106 suppressed the expression of BRCA2 and Rad51 at protein and mRNA levels in A549 cells**. (A) Effects of TAS106 on the expression levels of DNA repair-related proteins. Cells were treated with TAS106 (1 μM) for 12 or 24 h. After incubation, cell extracts were analyzed by Western blotting using specific antibodies. Actin was used as a loading control. (Left) Representative blots of three separate experiments are shown. (Right) Bands corresponding to each protein were quantified, and intensities of each protein were normalized to the intensity of actin. Data are expressed as mean ± SE of three experiments. (B) Effects of TAS106 on the mRNA expression levels of DNA repair-related proteins. Cells were treated with TAS106 (1 μM) for 12 or 24 h. After incubation, total RNA was isolated and the mRNA expression was analyzed by RT-PCR. Actin was used as an internal control. (Left) A representative image of three separate experiments is shown. (Right) Bands corresponding to each mRNA were quantified, and the intensity of each mRNA was normalized to the intensity of actin. Data are expressed as mean ± SE of three experiments.

To explore the mechanism of DSB inhibition by TAS106, we examined the effect of TAS106 on the expression levels of DSB repair-related proteins in A549 cells. As shown in Figure 4A, the treatment with 1 μM TAS106 for 24 h clearly suppressed the expression levels of BRCA2 and Rad51, which are the key proteins of the HR pathway. The expression levels of Mre11 and NBS1, which are constituent proteins of the MRN complex, were slightly reduced by TAS106. On the other hand, there was no obvious change in the expression levels of DNA-PKcs and Ku70, which are involved in the NHEJ pathway. It has been reported that TAS106 inhibits RNA synthesis and regulates the expression of its target proteins at the mRNA level [12,13]. Therefore, we examined the effect of TAS106 on the mRNA expression levels of DSB repair-related proteins using semiquantitative RT-PCR. Figure 4B shows that the treatment with 1 μM TAS106 suppressed the mRNA levels of BRCA2, Rad51, Mre11 and NBS1 in a time-dependent manner. However, the mRNA levels of DNA-PKcs and Ku70 were not changed. These tendencies had good correlation with the results of Western blot analysis. These results suggested that TAS106 suppressed HR-related proteins but not NHEJ-related proteins.

### Down-regulation of BRCA2 and Rad51 was maintained for 12 h after the removal of TAS106

**Figure 5:**
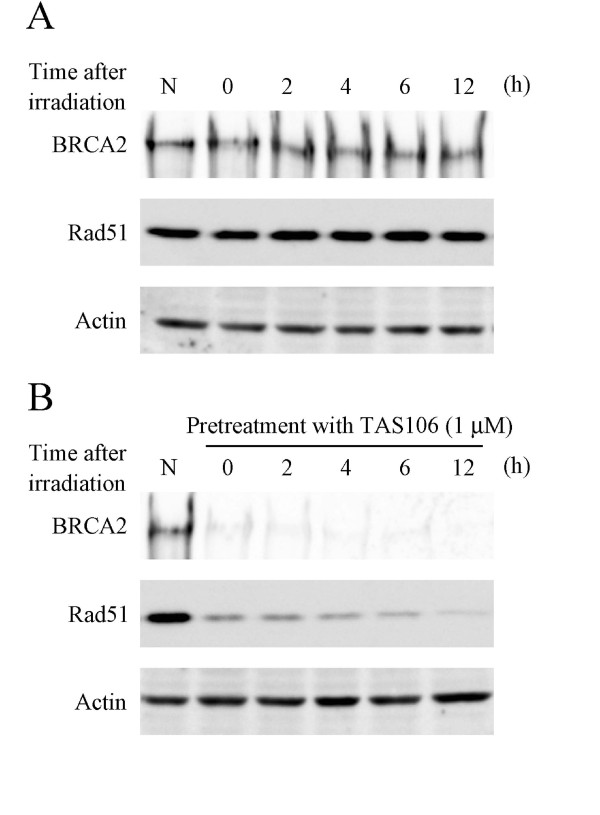
**Duration of the down-regulation of BRCA2 and Rad51 by TAS106 in A549 cells**. Cells were pretreated with or without TAS106 (1 μM) for 24 h. They were irradiated (10 Gy) and incubated for the indicated times in the absence of TAS106. After incubation, cell extracts were analyzed by Western blotting using specific antibodies. Actin was used as a loading control. (A) X-irradiation alone and (B) X-irradiation after pretreatment with TAS106. Representative blots of three separate experiments are shown.

For TAS106-induced down-regulation of BRCA2 and Rad51 to result in tumor radiosensitization, it needs to be maintained during the period of initial DSB repair after X-irradiation. Therefore, we assessed how long this inhibitory effect was prolonged after the removal of TAS106. A549 cells were treated with 1 μM TAS106 or vehicle for 24 h and replaced with fresh medium. After exposure to X-rays at 10 Gy, cells were collected at each time point. In cells exposed to X-irradiation alone there was no change in the expression levels of BRCA2 and Rad51 (Figure 5A). In contrast, TAS106 reduced the expression levels of BRCA2 and Rad51 and this persisted up to 12 h after X-irradiation (Figure 5B). These results suggested that TAS106 reduced the expression levels of HR-related proteins for a period long enough to inhibit the repair of radiation-induced DSBs after its removal.

### Down-regulation of BRCA2 is responsible for the radiosensitizing effect of TAS106

**Figure 6:**
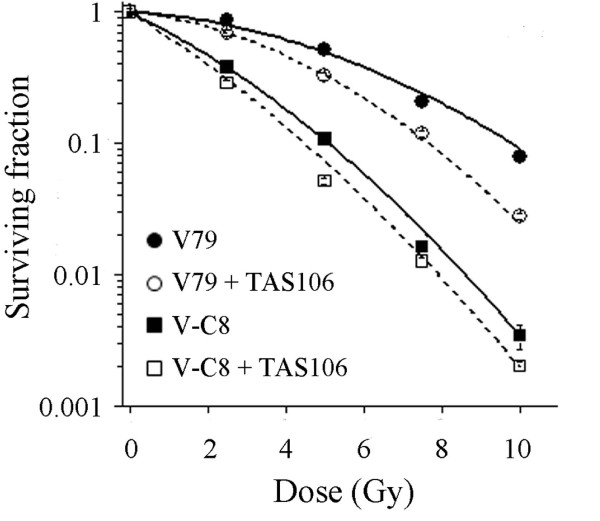
**Deletion of BRCA2 abrogated the radiosensitizing effect of TAS106**. Chinese hamster V79 and BRCA2-deficient V-C8 cells were pretreated with or without TAS106 (1 μM) for 6 h and its radiosensitizing effect was assessed by measuring clonogenic cell survival. *Closed circle*; X-irradiation for V79, *open circle*; X-irradiation + 1 μM TAS106 for V79, *closed square*; X-irradiation for V-C8, *open square*; X-irradiation + 1 μM TAS106 for V-C8. Data are expressed as mean ± SE of three experiments.

Based on the results described above, we hypothesized that the radiosensitizing effect of TAS106 was mainly due to abrogation of the HR pathway through the down-regulation of BRCA2. To test this hypothesis, we next compared the radiosensitizing effect of TAS106 in BRCA2-deficient V-C8 cells with that in parental V79 cells by clonogenic survival assay. It has been shown that the HR-pathway-mediated DNA damage repair is impaired in BRCA2-deficient V-C8 cells [23]. While a significant radiosensitizing effect by TAS106 was observed in V79 cells, the treatment with 1 μM TAS106 did not induce any radiosensitizing effect in V-C8 cells (Figure 6). In V79 cells, the pretreatment with 1 μM TAS106 reduced the D_10 _of the surviving fraction from 9.78 Gy to 7.67 Gy and its SER was 1.28. In V-C8 cells, the D_10 _of the surviving fraction was reduced from 5.09 Gy to 4.51 Gy and SER was 1.13. In addition, SER calculated from α value in V79 cells was also higher than that in V-C8 cells (Table 1). Therefore, this result suggested that down-regulation of BRCA2 by TAS106 suppressed the HR pathway of DSB repair, leading to the radiosensitizing effect.

## Discussion

The DNA repair pathway is a promising target for cancer radiotherapy because intrinsic DNA repair pathway enables tumor cells to survive by repairing radiation-induced DNA lesions. Therefore, there have been several studies aimed at radiosensitizing tumor cells by modulating DNA repair-related molecules. For example, the ATM inhibitor KU-55933 shows high specificity and induces radiosensitizing effects in tumor cells [24]. Gemcitabine and Gimeracil, which disrupt the metabolic pathway for nucleic acids, were also reported to have radiosensitizing effects through the inhibition of HR-mediated DSB repair [25,26].

We have previously reported that TAS106 enhances X-irradiation-induced apoptosis and reproductive cell death regardless of p53 status in tumor cells *in vitro *and *in vivo *[17,18]. The down-regulation of survivin, a key protein regulating apoptosis, and the abrogation of arrest at the G2/M phase by TAS106 are partly responsible for the enhancement of cell death [16]. Furthermore, TAS106 enhances X-irradiation-induced apoptosis even under hypoxic conditions through the down-regulation of HIF-1α [18]. Although TAS106 exhibits a clear radiosensitizing effect, its effect on DNA DSBs, which are the most lethal DNA lesions caused by X-irradiation, has not been evaluated yet. Therefore, we assessed the effect of TAS106 on the repair of radiation-induced DSBs.

In the present study, pretreatment with TAS106 enhanced radiation-induced cell death in tumor cell lines A549 and HEp-2 as well as the immortalized cell line V79 (Figure 2). To determine whether this radiosensitizing effect by TAS106 could be explained by the suppression of DNA repair capacity, we next investigated the effect of TAS106 on SLD repair. It has been reported that SLD repair reflects the cellular DSBs repair capacity mediated by the HR pathway [27,28]. As shown Figure 3A, TAS106 suppressed the SLD repair in A549 cells exposed to fractionated irradiation. In addition, the average numbers of radiation-induced γ-H2AX and 53BP1 foci in TAS106-pretreated cells were higher than in control cells up to 6 h after X-irradiation, as shown in Figure 3B and 3C. These results suggested that TAS106 suppressed DSB repair through inhibition of the HR pathway.

To clarify the molecular mechanisms of DSB repair inhibition by TAS106, we tested the effect of TAS106 on the expression levels of DSB repair-related proteins using Western blot analysis. The expression of NHEJ-related proteins, DNA-PKcs and Ku70, was not affected by TAS106. On the other hand, TAS106 suppressed the expression of the HR-related proteins BRCA2 and Rad51 (Figure 4A). Therefore, our results suggested that down-regulation of Rad51 and BRCA2 by TAS106 inhibited the HR pathway, leading to the radiosensitizing effect, as shown in Figure 2. RAD51 is a crucial component of the HR pathway and its down-regulation enhances radiosensitivity in tumor cells [29,30]. It has also been reported that deficiency or down-regulation of BRCA2 results in high sensitivity to X-irradiation due to insufficient DSB repair [10,31]. Recent studies reported that loss of HR capacity in BRCA2-deficient cells was restored by overexpression of wild-type Rad51 [32,33]. Therefore, although TAS106 reduced the expression levels of BRCA2 and Rad51, it might be possible that the down-regulation of Rad51 by TAS106 is more influential than that of BRCA2.

Down-regulation of BRCA2 and Rad51 was maintained up to 12 h after the removal of TAS106 and the following X-irradiation (Figure 5B). As shown in Figure 3B, DSBs were mostly repaired within 6 h after irradiation in control cells. Therefore, the maintenance of this down-regulation likely contributed to the inhibition of DSB repair by TAS106. Additionally, TAS106 reduced the mRNA levels of HR-related proteins in consistent with the result of Western blot analysis in A549 cells (Figure 4). These results suggested that TAS106 down-regulated these proteins transcriptionally, and supported the results that TAS106 inhibited RNA transcription by suppressing RNA polymerase II in previous studies [34-36].

To further support our findings, we compared the radiosensitizing effect of TAS106 in BRCA2-deficient V-C8 cells with that in parental V79 cells. TAS106 sensitized V79 cells, but not V-C8 cells, to X-irradiation (Figure 6). In addition, we examined the effect of TAS106 on the expression level of Rad51 in V79 and V-C8 cells. TAS106 did not change it in both cells (data not shown). These results suggested that the down-regulation of BRCA2, rather than Rad51, was primarily attributable to the suppression of cellular DNA repair capacity and the radiosensitizing effect in TAS106-treated cells.

## Conclusions

We have demonstrated that TAS106 suppresses the repair of radiation-induced DSBs and enhances radiosensitivity, which is mainly associated with the inhibition of HR pathway through the down-regulation of BRCA2. DSBs are the main target of cancer radiotherapy, and targeting inhibition of DNA repair is one approach to improve the efficiency of it. Therefore, TAS106 could be a good molecular candidate to achieve it, and the combination of TAS106 and X-irradiation may be an effective strategy for enhancing tumor cell death.

## Methods

### Reagents

RPMI 1640, DMEM and α-MEM medium were purchased from Invitrogen (Carlsbad, CA). Ham's F-10 medium and fetal bovine serum were purchased from Sigma-Aldrich (St. Louis, MO). 1-(3-C-ethynyl-β-D-ribo-pentofuranosyl) cytosine (TAS106) was synthesized as described elsewhere [11]. The following antibodies were used for Western blotting and immunostaining: anti-BRCA2 (Merck, Darmstadt, Germany), anti-NBS1 (Novus Biologicals, Littleton, CO), anti-Mre11, anti-53BP1 (Abcam, Cambridge, MA), anti-γ-H2AX (Millipore, Billerica, MA), anti-Rad51, anti-DNA-PKcs, anti-Ku70, anti-actin, HRP-conjugated secondary antibodies (Santa Cruz Biotechnology, Santa Cruz, CA), Alexa Fluor^® ^488 anti-mouse IgG and anti-rabbit IgG (Invitrogen). The chemiluminescence detection kit, Western Lightning^® ^Plus-ECL, was purchased from Perkin Elmer (Boston, MA).

### Cell culture, X-irradiation and drug treatment

Human lung carcinoma cell line A549, human larynx squamous carcinoma cell line HEp-2 and Chinese hamster fibroblast cell line V79 were grown in RPMI 1640, DMEM and α-MEM medium containing 10% fetal bovine serum at 37°C in 5% CO_2_, respectively. Chinese hamster fibroblast cell line V-C8, derived from V79, was grown in Ham's F-10 medium containing 10% fetal bovine serum. X-irradiation was performed with a Shimadzu PANTAK HF-350 X-ray generator (1.0 mm Al filter, 200 kVp, 20 mA, Shimadzu, Kyoto, Japan). Cells were treated with TAS106 for 6 h (V79 and V-C8) or 24 h (A549 and HEp-2) and subsequent X-irradiation was performed in the absence of TAS106.

### Clonogenic survival assay

Cells were seeded on 6-cm dishes and treated with TAS106 at the indicated concentrations for 6 h or 24 h. Then they were washed twice with PBS and replaced with fresh medium. Immediately after replacement, cells were exposed to X-rays and incubated for 7-14 days. Following this they were then fixed with methanol and stained with Giemsa solution (Sigma-Aldrich). Colonies containing more than 50 cells were scored as surviving cells. In A549 cells, 64, 48 and 29% of cells were alive at the concentration of 0.5, 0.75 and 1 μM TAS106, respectively. In addition, cell survivals after TAS106 treatment were 25, 98, and 35% at 0.1 μM for HEp-2, 1 μM for V79 and 1 μM for V-C8, respectively. Each surviving fractions were corrected using these cell survivals. The survival curves were fitted to a linear-quadratic model by data analysis software Origin Pro 7 (OriginLab Co. Northampton, MA).

Sublethal damage (SLD) repair capacity was measured as the increase of surviving cells after irradiation with a split dose at the indicated interval. Cells treated with 1 μM TAS106 for 24 h were washed twice with PBS and replaced with fresh medium. Immediately after replacement, cells were exposed to X-rays (2.5 Gy) and incubated for 0-12 h for the repair of SLD. At the indicated times, the cells were exposed to X-rays (2.5 Gy) again and incubated for 10 days.

### Immunofluorescent staining for γ-H2AX and 53BP1

At the indicated times after TAS106 treatment and X-irradiation, cells attached on glass coverslips were fixed in 4% paraformaldehyde/PBS for 30 min at room temperature. After being permeabilized with PBS containing 0.5% Triton X-100 for 5 min at 4°C, cells were treated with PBS containing 6% goat serum for 30 min at room temperature. Then they were incubated with the anti-*γ*-H2AX antibody at a 1:500 dilution or the anti-53BP1 antibody at a 1:1000 dilution in 3% goat serum overnight at 4°C. Cells were then incubated in the dark with the Alexa Fluor^® ^488-conjugated anti-mouse or anti-rabbit secondary antibody at a 1:500 dilution for 1.5 h. After incubation, they were counterstained with 300 nM 4',6'-diamidino-2-phenylindole (Invitrogen) for 5 min at room temperature. Coverslips were mounted with Prolong Gold antifade reagent (Invitrogen). Fluorescent microscopic analysis was performed using an Olympus BX50 microscope with reflected light fluorescence and foci were counted manually.

### SDS-PAGE and Western blotting

Cells were collected and lysed in lysis buffer (20 mM HEPES-NaOH [pH 7.4], 2 mM EGTA, 50 mM glycerophosphate, 1% Triton X-100, 10% glycerol, 1 mM PMSF, 10 μg/ml leupeptin, 10 μg/ml aprotinin and 10 μg/ml pepstatin). After centrifugation at 15,000 rpm for 15 min at 4°C, supernatants were collected. Three-fold concentrated Laemmli's sample buffer (0.1875 M Tris-HCl [pH 6.8], 15% β-mercaptoethanol, 6% SDS, 30% glycerol and 0.006% bromophenol blue) was added to the supernatant, and samples were boiled for 5 min. Proteins were separated by SDS-PAGE and transferred onto a nitrocellulose membrane (ADVANTEC Toyo, Tokyo, Japan). The membrane was probed with specific antibodies diluted with TBST (10 mM Tris-HCl [pH 7.4], 0.1 M NaCl and 0.1% Tween-20) containing 5% nonfat skim milk overnight at 4°C. After being probed with HRP-conjugated secondary antibodies, bound antibodies were detected with Western Lightning^® ^Plus-ECL.

### Semiquantitative reverse transcription-PCR (RT-PCR)

Total RNA was extracted and purified with an RNeasy Mini Kit (Qiagen, Hilden, Germany) according to the manufacturer's instructions. One microgram of RNA was reverse transcribed using the Reverse Transcription System (Promega Corporation, Madison, WI) and cDNA was amplified with GoTaq™ DNA Polymerase (Promega). The specific primer sequences for PCR were as follows: for BRCA2, 5'-CAAGCAGATGATGTTTCCTGTCC-3' and 5'-AGAACTAAGGGTGGGTGGTGTAGC-3'; for Rad51, 5'-TTTGGAGAATTCCGAACTGG-3' and 5'-AGGAAGACAGGGAGAGTCG-3'; for Mre11, 5'-CTTGTACGACTGCGAGTGGA-3' and 5'-TTCACCCATCCCTCTTTCTG-3'; for NBS1, 5'-AGAAATTGAGTTCCGCAGTTGTC-3' and 5'-GGGATTCTCATCTTAGCCAAAG-3'; for DNA-PKcs, 5'-ACACCATGTCCCAAGAGGAG-3' and 5'-AGCCTCAGGGCTTGTACTCA-3'; for Ku70, 5'-TATTTACGTCTTACAGGAGC-3' and 5'-GCATCTTCCTTTTATCATCA-3'; for actin 5'-GACCCAGATCATGTTTGAGACC-3' and 5'-GGTGAGGATCTTCATGAGGTAG-3'.

The PCR protocol was as follows: initial denaturation at 95°C for 2 min, followed by 31 cycles (BRCA2, Mre11, NBS1, DNA-PKcs, Ku70 and actin) or 40 cycles (Rad51) at 95°C for 1 min, annealing at 55°C (Ku70), 60.6°C (NBS1 and Mre11), 63°C (DNA-PKcs and actin), 64°C (Rad51) or 65°C (BRCA2) for 1 min and extension at 72°C for 1 min. The final extension was performed by incubation at 72°C for 5 min. The PCR products were subjected to agarose gel electrophoresis and visualized using ethidium bromide (Sigma-Aldrich).

## List of abbreviations

ATM: ataxia telangiectasia mutated; DSBs: DNA double strand breaks; TAS106: 1-(3-C-ethynyl-β-D-ribo-pentofuranosyl)cytosine (ECyd); HIF-1α: hypoxia inducible factor-1α; HR: homologous recombination; MRN: Mre11-Rad50-NBS1; NHEJ: non-homologous end joining; SLD: sub-lethal damage; ssDNA: single strand DNA; UCK: uridine/cytidine kinase.

## Competing interests

The authors declare that they have no competing interests.

## Authors' contributions

SM and HY performed the research, analyzed the data, and drafted the manuscript. ME helped with cell culture and Western blotting techniques. MM prepared the V-C8 cells used in these studies. MF, YY and AM synthesized TAS106 used in these studies. TY and OI designed the research, interpreted the data. All authors approved the final version of the manuscript.
